# Under the Hood: Using Computational Psychiatry to Make Psychological Therapies More Mechanism-Focused

**DOI:** 10.3389/fpsyt.2020.00140

**Published:** 2020-03-18

**Authors:** Akshay Nair, Robb B. Rutledge, Liam Mason

**Affiliations:** ^1^Department of Neurodegenerative Disease, UCL Institute of Neurology, University College London, London, United Kingdom; ^2^Max Planck UCL Centre for Computational Psychiatry and Ageing Research, Berlin, Germany; ^3^Wellcome Centre for Human Neuroimaging, University College London, London, United Kingdom; ^4^Research Department of Clinical, Educational and Health Psychology, University College London, London, United Kingdom

**Keywords:** psychological therapies, decision-making, computational psychiatry, computational neuroscience, CBT (cognitive-behavioral therapy), reinforcement learning (RL), mechanisms, reward (healthcare)

## Abstract

Psychological therapies, such as CBT, are an important part of the treatment of a range of psychiatric disorders such as depression and anxiety. There is a growing desire to understand the mechanisms by which such therapies effect change so as to improve treatment outcomes. Here we argue that adopting a computational framework may be one such approach. Computational psychiatry aims to provide a theoretical framework for moving between higher-level psychological states (like emotions, decisions and beliefs) to neural circuits, by modeling these constructs mathematically. These models are explicit hypotheses that contain quantifiable variables and parameters derived from each individual's behavior. This approach has two advantages. Firstly, some of the variables described by these models appears to reflect the neural activity of specific brain regions. Secondly, the parameters estimated by these models may offer a unique description of a patient's symptoms which can be used to both tailor therapy and track its effect. In doing so this approach may offer some additional granularity in understanding how psychological therapies, such as CBT, are working. Although this field shows significant promise, we also highlight several of the key hurdles that must first be overcome before clinical translation of computational insights can be realized.

## Introduction

There is growing recognition that, to move forward, the field of psychological therapy needs to return to its scientific roots and become more mechanism focused. A Lancet commission for improving psychological therapies urged greater synergy between basic and clinical research (1). In this paper, we argue that considering psychological therapies from a computational perspective may be one such approach to achieve this aim.

The emerging field of computational psychiatry aims to provide a theoretical framework for moving between higher-level psychological states (including emotions, decisions and beliefs) to neural circuits, by modeling these constructs mathematically (2, 3). Here, we do not cover the breath of research in computational psychiatry and direct interested readers to other recent reviews (3, 4). Instead, the focus of this piece is how a computational framework may translate to psychological therapies by allowing us to get closer to the generating mechanisms of symptoms and distress (5, 6). For a consideration of how these frameworks might provide insight into the generating and maintaining processes targeted by psychological therapies, the reader is referred to (5). In this article, we primarily focus on the application of “reinforcement learning” models (7) to understanding and evaluating cognitive behavior therapy (CBT) specifically. There are a wide range of psychological treatment modalities, such as psychodynamic therapy (8) and humanistic (9) approaches. There are also many approaches derived from or compatible with computational modeling, including active inference (10) and perceptual control theory (PCT) (11, 12) which make different predictions about the relationship between internal states and behavior [for example in PCT, the control of sensory input through behavior, see (11)]. It is beyond the scope of this article to cover them all, and we focus on reinforcement learning and CBT as examples, as two of the most widespread frameworks. We do not necessarily advocate for these two frameworks above others; rather, we use them as salient examples of how computational theory can help us better to understand how psychological therapy works. We also discuss some of the challenges that currently limit the translation of computational psychiatry into clinical practice.

### What Is Computational Psychiatry?

A central goal of CBT is to support patients in moving toward their goals, which will typically involve helping patients to adapt their beliefs and behavior to their current environment (13). Put in computational terms, such therapies, in effect, alter the mapping between *states* the person is in, for example an anxiety-provoking situation, like giving a presentation, and *actions*, such as staying or escaping. In computational terms, this mapping between states and actions is known as the *policy* (7). By helping patients adapt their beliefs and behavior, psychological therapies can be understood as helping patients adopt new policies. For example, consider a musician with depression reporting that they now get less pleasure (or *reward*) from a past-time that they used to enjoy, such as playing the guitar, and so have stopped entirely. Here, a possible psychological mechanism maintaining their depression would be behavioral avoidance (14). That is, a lack of positive reinforcement from their environment and/or negative reinforcement of the avoidant behavior (e.g., not playing the guitar serves to avoid experiencing negative emotions of feeling upset and ashamed whenever they don't play the guitar perfectly).

**Figure 1:**
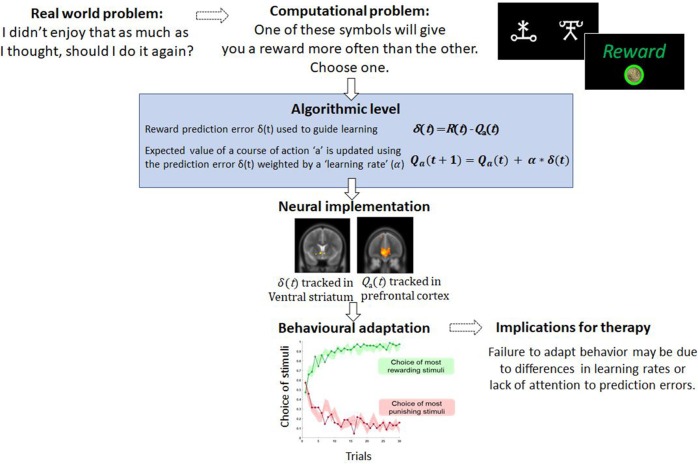
An example of a computational problem—learning which actions are the most and least rewarding. In typical tasks, participants choose between stimuli which differ in terms of reward probabilities. Through trial and error participants learn to choose and to avoid the best and worst options, respectively. This type of behavior is well-captured by simple computational models like the one described and variables from these models, such as the prediction error, correlated with brain activity. For therapy, failure to learn from rewarding or punishing experiences may limit the capacity to change behavior.

Algorithmically, this *prediction error*, the difference between reward and expectation, is used to update the “value” of that action for the future. In this case, the *value* of playing the guitar will reduce, so that in the future when trying to decide what to do, it is much less likely that playing guitar will be chosen because the expected value has fallen (Figure 1). As such, the patient's *policy* has changed. Successful behavioral therapy would seek to reverse this. Within computational models, this updating is typically controlled by a parameter known as the *learning rate*. Variability in the learning rate parameter reflects how sharply the value of actions, such as choosing to play guitar, are changed by recent prediction errors.

### Can Computational Parameters Be Used to Measure Change During Psychological Therapy?

If CBT results in changes in policy, then computational parameters such as the learning rate, should determine the rate of this change and therefore, may be an early predictor of treatment response. Indeed, if parameters control a process that generates or maintains psychiatric symptoms, then changes to these parameters could precede and mediate subsequent improvement of those symptoms. An analog from pharmacotherapy is the finding that the clinical benefit of antidepressant medication, which is typically on the order of weeks (15), may actually be preceded by a normalizing effect on the neural processing of negative emotional information that is evident on the order of hours (16). As such, change in computational model parameters may also serve as useful secondary outcomes for evaluating the effectiveness of psychological therapy. For example, in patients with anxiety who have measurable differences in learning rates as compared to controls (17), change in these parameters may suggest successful psychological intervention even if symptoms have not yet reached remission. This is analogous to the use of biomarkers in clinical trials being used as secondary outcomes—for example, changes in amyloid-beta measures in clinical trials for Alzheimer dementia may suggest possible treatment efficacy even if no change is seen in cognitive scores (18). It has previously been proposed that outcomes in psychological therapy could be formally modeled, using *dynamic systems theory*, for example (19, 20). As compared to these proposals, computational modeling parameters from reinforcement learning models would serves to model the underlying computational process driving change rather than the complex dynamics of psychotherapy. This is, of course, dependent on identifying computational differences that track the relevant state or trait features that psychological therapy is aiming to target. From pharmacology studies it can be shown than administering drugs that act on dopamine and serotonin neurotransmitter systems, for example, can alter fitted computational parameters in behavioral tasks (21, 22). The same may be true for efficacious psychological therapies.

The computational approach is also appealing because variables derived from algorithmic models have been shown to be tracked by the brain (23). This is valuable because neurobiological measures provide objective measures of the processes that generate behavior which are often not amenable to accurate self-report (1). Moreover, there is amassing empirical evidence that psychological therapy leads to reorganization at the neural level. For example, longitudinal functional MRI studies of CBT identify a strengthening of connections between prefrontal cortex and limbic regions [consistent with contextualizing and regulating emotion and potential threat (24, 25), and that the extent of these CBT-led changes may predict the degree of remission experienced several years post-therapy (26)]. In tasks that involve learning and the updating of beliefs and behavior, *reinforcement learning* algorithms not only account for behavior but also predict regional brain activity. Numerous fMRI studies have reported that the *prediction error* signal, described above, correlates with the activity in the ventral striatum and frontal cortices in fMRI studies of instrumental learning (23) (Figure 1), and there is recent evidence that these signals predict response to CBT (27). The expected value of a chosen option has also been shown to correlate with activity in the medial prefrontal cortex (28). Outside of learning tasks, other models which consider costs show that effort and net-value may be coded in different brain regions such as the cingulate cortex (29). If these computational parameters and variables from models are associated with psychological recovery, this approach may offer deeper localization of the therapy effects. Furthermore, recent movements in CBT have focused attention away from disorder-specific interventions to underlying psychological processes appearing across multiple disorders (30, 31). This newer approach is arguably a better fit with computational modeling of brain and behavior as the role of computational parameters in determining decision-making or behavior is not limited by diagnosis. Largely in this article we have focused on parameters that change with clinical state. However, variance in these parameters when patients are well may be a readout of underlying processes which put patients at risk of mental health difficulties.

### Can Computational Parameters Inform Formulation and Tailor Delivery of Psychological Therapy?

Computational models make several testable predictions relevant to understanding the variance in the efficacy of psychological therapies. Firstly, change techniques will be less effective for those patients who struggle to update the value of actions based on new evidence (i.e., those with lower learning rates for that target belief or behavior). Secondly, more targeted approaches that increase learning rates for therapy-guided action-outcome pairs may increase the effectiveness of interventions (by fostering new learning and updating policies). A key factor determining the success of cognitive-behavioral techniques such as behavioral experiments, in which patients test out what happens when in a feared situation, is whether their prediction about what will happen has been clearly operationalized (32). In the current framework, this step ensures that the prediction error generated if the outcome differs from what was feared is then optimally attended to and utilized to update future expectations about similar situations. Indeed, attention to prediction errors has been shown to be a key process for efficient learning and belief updating (33). Therefore, a third prediction would be that adding preparatory work that promotes attention to prediction errors will increase the effectiveness of the separate learning-based intervention. Behavioral interventions for depression, for example, assume that low mood is maintained by a reduction in rewarding experiences in a person's life and aim to bring the client back into contact with meaningful rewarding experiences. An important component involves the client making formal predictions about how enjoyable, or unenjoyable, an activity will be and re-rating that with the actual level of enjoyment experienced (14). This process increases the chances that a prediction error is computed and utilized to update beliefs.

**Figure 2:**
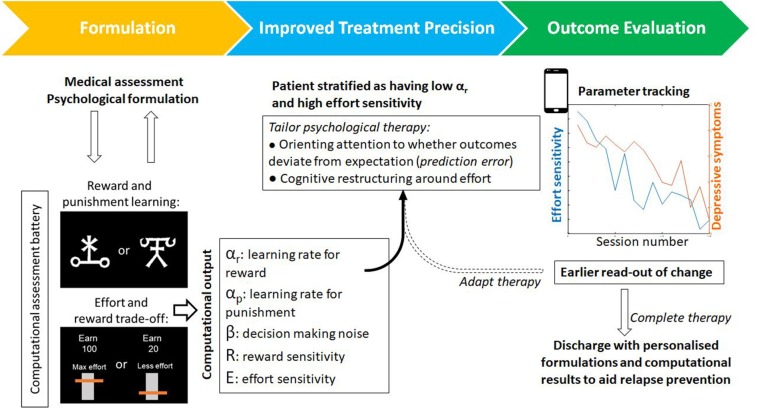
An example of a patient journey through psychological therapy that incorporates computational assessments. Alongside psychological formulation, task-based assessments output individual parameters for a range of computational processes–such as reward learning or effort discounting. From this analysis, a tailored computational profile could be used to tailor components of therapy (for example attention and learning during behavioral activation versus cognitive restructuring of beliefs around effort). These computational parameters provide objective markers for evaluating change and, by virtue of being closer to the underlying mechanisms that generate or maintain symptoms, may provide an earlier readout of change that precedes symptom improvement. Tasks exist in adapted forms for use online or on smart devices that may also improve patient access and engagement.

Computational work also suggests that psychological therapies could be tailored to individual patients. Recent work has shown that prediction errors come in many shapes and sizes. An action could also vary not only in the amount of reward it yields but in the estimated effort, or other costs, needed to obtain reward. There is evidence that estimations about any of these aspects may go awry when someone is depressed (34). Behavioral activation often involves the client quantifying how rewarding an activity will be (reward predictions), but recent work shows that the brain also computes *effort* prediction errors used to learn the costs associated with different states (35). These effort prediction errors could also be harnessed for the purpose of learning and updating as described above (Figure 2). As with reward prediction errors, learning rate may also vary between patients. Patients could therefore be stratified therefore not only on reward-based parameters but differences in cost-based parameters like effort sensitivity and effort learning rates. These parameters could be used to tailor the cognitive-behavioral change techniques that are deployed.

## Recommendations and Future Directions

Although this approach holds significant promise, we lack concrete examples of computational psychological therapy. There is promising work for example in anxiety, implicating the failure to adaptively modulate learning rate (36) that nonetheless currently falls short of clinical translation. The field also faces a number of hurdles which limit its application into clinical practice—these include reproducibility, generalizability, and scalability (Box 1). Computational parameters are estimated by fitting a model to behavior generated in bespoke behavioral tasks. In its nascent state, much computational research is currently focused on expanding current knowledge often with novel tasks and new models of behavior. For computational modeling to be useful in clinical practice, equal focus should be placed on pragmatic concerns such as generating reproducible findings and increasing the test-retest reliability of task performance and parameter estimation. For computational parameters to act as secondary outcomes in therapeutic trials they will be required to show stability over time in relevant control groups. Test-retest reliability of computational parameters appears currently modest (38), reducing the power of clinical trials where they could offer greater granularity to detect changes in cognitions and behavior that are relevant to treatment efficacy. Furthermore, the use of bespoke tasks in individual studies limits interpretability and evidence synthesis (including meta-analysis across studies). Avoiding this confusion is especially important when seeking to synthesize effect of a treatment on an outcome, such as the effect of CBT for anxiety on the learning rate in volatile environments.

Box 1 **Road blocks for integrating computational approaches to understanding psychological therapy**

1. Reliability. Whilst computational model parameters show promise, their test-retest reliability for specific tasks often remains to be established. In order to reap the benefits of more objective and direct measures of neuro-computational processes, extensive validation of the stability of tasks and model parameters over time is needed. Only computational parameters that show good test-retest reliability can they be used to track clinical state and evaluate the effectiveness of interventions.
2. Generalizability. Even once stable task measures are derived, a major gap to be filled is to confirm that they are predictive of real-life phenomena. For example, model parameters purporting to measure mood instability within an experimental setting should predict real-life mood fluctuations experienced by patients (for example via experience sampling). A second requirement is that if change in these task-based parameters is effected by some intervention, it should translate to benefits in real-life symptoms and functioning. This is analogous to the challenges faced by approaches involving cognitive training (37).
3. Scalable and easy to implement. Unlike other biomarker approaches including those which require neuroimaging technology, computational approaches provide inexpensive and practical measures that are collected behaviorally but grounded in neurobiology. They lend themselves to convenient online and smartphone-based data collection. This makes them more practical and far less expensive than fMRI for the purpose of patient stratification, treatment selection and other clinical decision-making. They also lend themselves to longitudinal “self-assessment” for the purpose of symptom monitoring and/or treatment evaluation.

Second, although parameters from models govern behavior, the degree to which they predict behavior outside of carefully controlled experimental conditions remains to be robustly established. Ideally, computational model parameters should capture real-life phenomena in ecological settings, and changes seen in controlled settings must generalize between the two. There does not appear to be good support for this at present (39). Others have argued the need for ecologically valid tasks and models (40) and this argument becomes stronger when wanting to demonstrate functional improvement following psychological therapy. Returning to the example of the depressed patient who no longer enjoys playing the guitar, computational parameters from a reward task should ideally predict not only abstract task performance but the patient's ability to return to their hobbies as they find them more enjoyable. Third, achieving this may also benefit from data collection in real-life settings, since they are closer to the environment which neural circuits should be tuned to and where symptoms arise. As alluded to above, unlike other biomarker approaches which require neuroimaging technology or biofluids, computational approaches can provide inexpensive and practical measures that are collected behaviorally but grounded in the underlying neurobiology. They lend themselves to convenient online and smartphone-based data collection. This makes them more practical and far less expensive than fMRI for the purpose of patient stratification, treatment selection and other clinical decision-making. They also lend themselves to longitudinal “self-assessment” for the purpose of symptom monitoring and treatment evaluation. There are already notable examples of the scalability of this approach for objective smartphone-based measurements of mood and decision-making, including in the clinical population (2, 41, 42). This approach also allows task-derived parameters to be validated alongside the real-life phenomena they supposedly capture, for example by tracking mood and associated measures of activity such as step count (43).

In summary, we argue that the inclusion of the computational characterization of behavior for patients undergoing psychological therapies offers a number of unique advances. Firstly, it is a principled theoretical framework to bridge psychological constructs with neural circuitry and function via mathematical models of cognitive processes. These models are explicit testable hypotheses with quantifiable parameters which govern individual differences in learning and behavior. As such, these models can be used to assess the impact of psychological processes on deeper processes such as learning. Finally, parameters from computational models may act as secondary outcome measures predicting psychological recovery that may precede behavioral or changes in symptom levels (Figure 2). There has been much recent excitement about the possibility of neuroimaging biomarkers for patient stratification (44). It remains to be seen whether computational biomarkers will achieve the same level of performance as neuroimaging biomarkers, but given that the costs can be more than a 100-fold lower, computational approaches hold considerable promise to improve psychological therapies. There remain a number of challenges. In order to be beneficial to treatment trials, computational tasks should be standardized and their parameter estimates should be stable in the absence of intervention and predictive of real-world behavior. If there is sufficient appetite from practitioners and patients, to adopt computational frameworks, we are optimistic that these limitations will be addressed and adopting a computational approach to the study of psychological therapies will allow us to peer under the hood and to improve our therapies for patients.

## Author Contributions

LM and AN contributed the conceptual aspects of the paper. All authors contributed to writing the manuscript.

### Conflict of Interest

The authors declare that the research was conducted in the absence of any commercial or financial relationships that could be construed as a potential conflict of interest.
